# Ddc2 Mediates Mec1 Activation through a Ddc1- or Dpb11-Independent Mechanism

**DOI:** 10.1371/journal.pgen.1004136

**Published:** 2014-02-20

**Authors:** Amitava Bandhu, John Kang, Kenzo Fukunaga, Greicy Goto, Katsunori Sugimoto

**Affiliations:** 1 Department of Microbiology and Molecular Genetics, New Jersey Medical School, Rutgers, The State University of New Jersey, Newark, New Jersey, United States of America; University of California San Francisco, United States of America

## Abstract

The protein kinase Mec1 (ATR ortholog) and its partner Ddc2 (ATRIP ortholog) play a key role in DNA damage checkpoint responses in budding yeast. Previous studies have established the model in which Ddc1, a subunit of the checkpoint clamp, and Dpb11, related to TopBP1, activate Mec1 directly and control DNA damage checkpoint responses at G1 and G2/M. In this study, we show that Ddc2 contributes to Mec1 activation through a Ddc1- or Dpb11-independent mechanism. The catalytic activity of Mec1 increases after DNA damage in a Ddc2-dependent manner. In contrast, Mec1 activation occurs even in the absence of Ddc1 and Dpb11 function at G2/M. Ddc2 recruits Mec1 to sites of DNA damage. To dissect the role of Ddc2 in Mec1 activation, we isolated and characterized a separation-of-function mutation in *DDC2*, called *ddc2-S4*. The *ddc2-S4* mutation does not affect Mec1 recruitment but diminishes Mec1 activation. Mec1 phosphorylates histone H2A in response to DNA damage. The *ddc2-S4* mutation decreases phosphorylation of histone H2A more significantly than the absence of Ddc1 and Dpb11 function does. Our results suggest that Ddc2 plays a critical role in Mec1 activation as well as Mec1 localization at sites of DNA damage.

## Author Summary

When DNA replication is blocked and DNA damage occurs, checkpoints arrest the cell cycle in eukaryotic cells, allowing DNA replication and repair to take place. The major regulators of the DNA damage checkpoint response are the phosphoinositide 3-kinase (PI3K)-related protein kinases, including ATM and ATR. In budding yeast, ATM and ATR correspond to Tel1 and Mec1, respectively. ATM/Tel1 acts in response to double-strand breaks. By contrast, ATR/Mec1 recognizes many different types of DNA damage. Mec1 forms a complex with Ddc2 (ATRIP ortholog) that recruits Mec1 to sites of DNA damage. We isolated a *ddc2* mutation that confers defects in DNA damage responses but does not impair Mec1 recruitment. We found that the catalytic activity of Mec1 increases in a Ddc2-dependent manner after DNA damage. Previous studies have demonstrated that Mec1 activation occurs through two independent pathways at G1 and G2/M: one pathway through Ddc1, a subunit of the checkpoint clamp and the second through Dpb11, the TopBP1 ortholog. We found that Mec1 activation occurs at least in part independently of Ddc1 and Dpb11. Our results suggest that Ddc2 stimulates Mec1 by a different mechanism than Ddc1 or Dpb11.

## Introduction

The DNA damage response pathways coordinate DNA repair, replication and cell-cycle progression to maintain genome integrity [1], [2]. Initiation of DNA damage responses requires two evolutionarily conserved phosphoinositide 3-kinase (PI3K)-related protein kinases: ATM and ATR. While ATM responds primarily to DNA double-strand breaks (DSBs), ATR recognizes various types of DNA lesions with single-stranded DNA (ssDNA) [2], [3]. In the budding yeast *Saccharomyces cerevisiae*, Mec1, the ATR ortholog, plays a critical role in the DNA damage response throughout the cell-cycle [1], [4]. Mec1 forms a stable complex with Ddc2 (ATRIP in human) [5]–[7], which recruits Mec1 to sites of DNA damage by interacting with replication protein A (RPA)-coated ssDNA [8]–[11]. Once recruited to DNA lesions, Mec1 phosphorylates the C-terminal tail of histone H2A [12]. Phosphorylated histone H2A creates a DNA damage mark similar to phosphorylated histone H2AX (γH2AX) in human [4], [13]. Phosphorylation of histone H2A, together with constitutive methylation of histone H3, promotes the recruitment of the checkpoint mediator, Rad9 [14], [15]. In turn, Mec1 phosphorylates Rad9 at sites of DNA damage [16], [17]. Phosphorylated Rad9 creates a docking site for the effector kinase, Rad53 (Chk2 in human) [18]–[20]. The Rad9-Rad53 interaction promotes Rad53 autophosphorylation and allows Mec1 to phosphorylate Rad53, leading to hyperphosphorylation and activation of Rad53 [20]–[23].

To execute full activation of Rad53, Mec1 collaborates with the checkpoint clamp complex and its loader as well [4]. The checkpoint clamp complex, structurally related to PCNA, consists of Ddc1, Mec3 and Rad17 (Rad9, Hus1 and Rad1 in human) [24], [25]. The checkpoint clamp loader is composed of Rad24 (Rad17 in human) and the four small RFC subunits [25]–[27]. The Rad24-RFC complex recognizes and loads the Ddc1-Mec3-Rad17 complex at junctions between ssDNA and double-stranded DNA on partial duplex DNA [25], [28]. Co-accumulation of Mec1 and Ddc1 at sites of DNA damage is essential for checkpoint activation [29], [30]. Supporting this view, tethering of both Ddc1 and Ddc2 on chromatin is sufficient for Rad53 phosphorylation even in the absence of DNA damage [31]. Once loaded at sites of DNA damage, the Ddc1-Mec3-Rad17 complex stimulates the association of Dpb11 (TopBP1 in human) with Mec1 [32]. Ddc1 and Dpb11 play a crucial role in DNA damage checkpoints at G1 and G2/M [33]–[36]. *In vitro* reconstitution studies have shown that Ddc1 or Dpb11 increases the catalytic activity of Mec1 [34], [35], [37]–[40]. These observations have established the model in which Ddc1 and Dpb11 govern the checkpoint pathway at G1 and G2/M by directly activating Mec1. In parallel with Ddc1 and Dpb11, Dna2 controls DNA damage and replication checkpoints specifically in S-phase by directly activating Mec1 [41]. Interestingly, all Ddc1, Dpb11 and Dna2 proteins utilize the unstructured domains with aromatic amino acid residues (Trp or Tyr) to increase the catalytic activity of Mec1 [34], [35], [41]. Moreover, the activation domain of Ddc1 is functionally replaceable with that of Dna2 [41]. Thus, Ddc1, Dpb11 and Dna2 appear to activate Mec1 through a similar mechanism.

In this study we show that Ddc2 stimulates Mec1 activity by a different mechanism than Ddc1 and Dpb11 at G2/M. Dpb11 possesses the Mec1 activation domain at the C-terminus. We used a strain expressing a C-terminal truncation mutation of Dpb11 encoded by the *dpb11-1* mutant allele and the deletion of *DDC1*, and found that Mec1 activation occurs after DNA damage even in *ddc1Δ dpb11-1* mutants at G2/M. However, Mec1 activation was fully dependent on Ddc2. Ddc2 plays an essential role in the recruitment of Mec1 to DNA lesions. To dissect the role of Ddc2 in Mec1 activation, we screened mutations in *DDC2*, which cause defects in DNA damage response but do not affect Mec1 recruitment. We characterized one of the separation-of-function mutations, *ddc2-S4*. The *ddc2-S4* mutation, unlike the *ddc1Δ dpb11-1* mutation, abolished damage-induced Mec1 activation. Cells carrying the *ddc2-S4* mutation showed more significant defects in damage-induced phosphorylation of histone H2A than *ddc1Δ dpb11-1* mutants at G2/M. These results are consistent with the model in which Ddc2 controls Mec1 activation by a different mechanism than Ddc1 or Dpb11.

## Results

### Requirement of Ddc2 for damage-induced Mec1 activation

**Figure 1:**
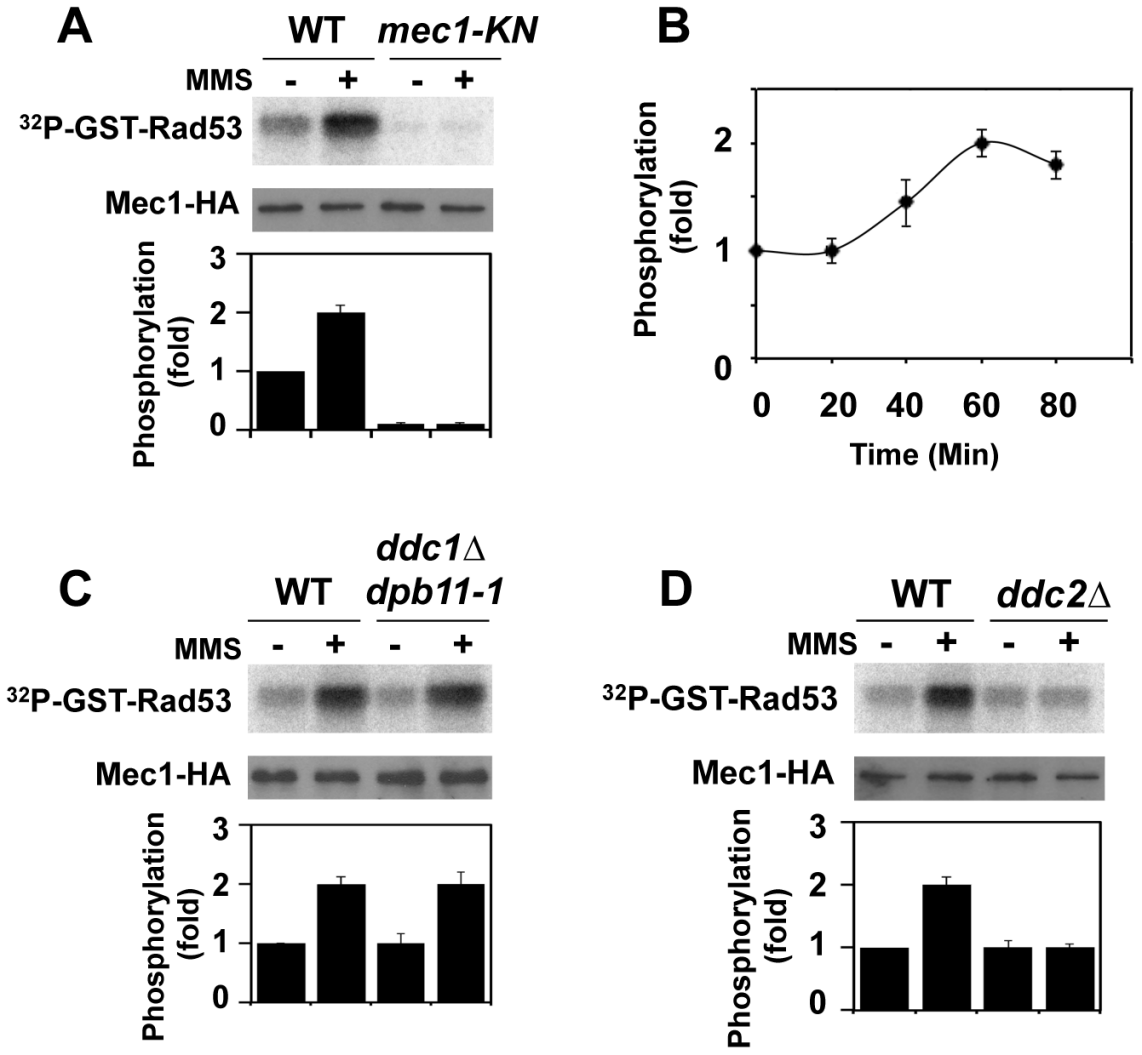
Regulation of protein kinase Mec1 after DNA damage. (A) Mec1 activation after MMS treatment. Cells expressing Mec1-HA (KSC1635) or Mec1-KN-HA (KSC1645) were cultured at 25°C and incubated with nocodazole to arrest at G2/M. Cells were then either mock treated (−) or treated (+) with 0.05% MMS for 1 hr. Immunoprecipitated Mec1-HA was subjected to the *in vitro* kinase assay using GST-Rad53, a recombinant protein purified from *E. coli*, as a substrate. Incorporation of ^32^P into GST-Rad53 was monitored by phosphorimager. The amounts of Mec1 or Mec1-KN in the immunoprecipitates were determined by immunoblotting with anti-HA antibody. Phosphorylation of GST-Rad53 was normalized to that observed with immunoprecipitated mock treated wild-type Mec1. Relative phosphorylation is determined from three independent experiments. (B) Mec1 activation kinase after incubation with MMS for various lengths of time. Cells were treated with MMS for indicated lengths of time and were subjected to the *in vitro* kinase assay as in A. (C) Effect of *ddc1Δ dpb11-1* mutation on Mec1 activation. Wild-type (KSC1333) and *ddc1Δ dpb11-1* (KSC3130) cells expressing Mec1-HA protein were subjected to the *in vitro* kinase assay as in A. (D) Effect of *ddc2Δ* mutation on Mec1 activation. Wild-type (KSC1635) and *ddc2Δ* (KSC1636) cells expressing HA-Mec1 protein were subjected to the *in vitro* kinase assay as in A.

We examined whether Mec1 catalytic activity is increased after DNA damage. Activation of the Mec1 signaling pathway is controlled in a cell-cycle dependent manner [34]–[36]. We thus monitored Mec1 kinase activity in G2/M-arrested cells (Fig. 1A). Cells expressing HA-tagged Mec1 (Mec1-HA) protein were arrested with nocodazole and then treated with methylmethane sulfonate (MMS). As a negative control cells expressing the kinase negative version of Mec1-HA (Mec1-KN-HA) were examined. HA-Mec1 proteins were immunoprecipitated from cell extracts with anti-HA antibodies, and immunoprecipitates were subjected to an *in vitro* kinase assay. As a substrate we used the GST-fusion protein with the C-terminus of Rad53 (GST-Rad53) [42]. Phosphorylation of the Rad53 C-terminus is critical for Rad53 activation [23]. Phosphorylation of GST-Rad53 was detected with immunoprecipitates from untreated cells, but the level increased after MMS treatment. No phosphorylation was detected with Mec1-KN-HA, indicating that the observed phosphorylation depends on Mec1 kinase activity. Mec1 was activated two-fold after exposure to 0.05% MMS for 1 hr (Fig. 1B). Similar activation was detected even if cells were treated with phleomycin (data not shown).

The unstructured C-terminal tails of Ddc1 and Dpb11 increase Mec1 kinase activity and control the Mec1-Rad53 checkpoint pathway at G2/M [34], [35], [38]. We next addressed whether Mec1 is activated after DNA damage in a Ddc1- or Dpb11-dependent manner (Fig. 1C). Because *DPB11* is essential for cell proliferation, we used the C-terminal truncation mutation in DPB11, *dpb11-1*, which confers defects in checkpoint activation at G2/M [34], [35], [43]. We examined the effect of *ddc1Δ dpb11-1* mutation on Mec1 kinase activity before and after MMS treatment (Fig. 1C). DNA flow cytometry analysis confirmed that *ddc1Δ dpb11-1* mutants were largely arrested with G2/M DNA contents after nocodazole treatment (Fig. S1). MMS-induced Mec1 activation was observed in *ddc1Δ dpb11-1* mutants, indicating that Mec1 activation occurs after DNA damage independently of Ddc1 and Dpb11. Our assay was not able to detect Ddc1- or Dpb11-dependent Mec1 activation, but this observation does not contradict the idea that Ddc1 or Dpb11 directly activates Mec1 [34], [35], [38] (see below). Mec1 forms a stable complex with Ddc2 [5]–[7]. We examined the effect of *ddc2Δ* mutation on Mec1 activity before and after DNA damage (Fig. 1D). *DDC2* is essential for cell proliferation [5]–[7]. Since the lethality of *ddc2* disruption is suppressed by *sml1* mutation [5]–[7], we examined the effect of the *ddc2Δ* mutation in a *sml1*Δ background. The *ddc2Δ* mutation did not impair basal kinase activity of Mec1, but abolished increase in catalytic activity after MMS treatment.

### Identification of Ddc2 domain that is involved in Mec1-Ddc2 interaction or DNA damage responses

**Figure 2:**
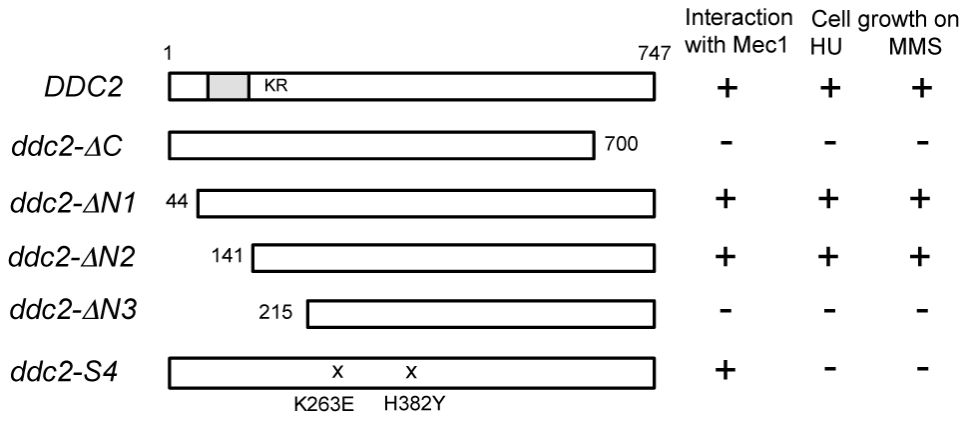
Identification of the Ddc2 region required for Mec1 interaction and DNA damage response using the modified two-hybrid assay. The two-hybrid tester *ddc2Δ* strain (KSC2077) carrying pBD-MEC1(2-2368) was transformed with pAD-DDC2, pAD-DDC2-ΔC, pAD-DDC2-ΔN1, pAD-DDC2-ΔN2, pAD-DDC2-ΔN3 or pAD-DDC2-S4. The coiled-coil domain (amino acid 68–138) is indicated as a gray bar and the KKRK sequence (amino acid 177–180) is shown as KR. The *ddc2-S4* mutation carries two substitution mutations (K263E and H382Y). Transformants were streaked on an SD-Ura-Leu-His plate containing 1 mM AT, YEPD plate containing 1 mg/ml HU or 0.005% MMS. Interaction with Mec1 and DNA damage sensitivity were assessed by cell proliferation on the respective plates.

One explanation for the above results is that Ddc2 mediates Mec1 activation independently of Ddc1 or Dpb11. However, accumulation of Mec1 at sites of DNA damage might be sufficient for Mec1 activation, since Ddc2 plays a key role in recruiting Mec1 to DNA lesions [8], [9], [11]. We therefore sought separation-of-function mutation(s) in *DDC2*, which confer defects in damage-induced Mec1 activation but not in Mec1 recruitment. To narrow the target area for mutagenesis, we first identified domain(s) in Ddc2 that are necessary for DNA damage response or Mec1-Ddc2 interaction in a modified two-hybrid system (Fig. 2). Mec1-Ddc2 interaction was monitored using the binding domain (BD)-MEC1 fusion and the activation domain (AD)-DDC2 fusion [7]. To assess DNA damage response function, we introduced a *ddc2Δ* mutation in the two-hybrid tester strain. Cells carrying a *ddc2Δ* mutation are sensitive to DNA damaging agents including hydroxyurea (HU) or MMS. The full-length AD-DDC2 construct supported both Mec1-Ddc2 interaction and cellular response to HU or MMS treatment (Fig. 2). The C-terminal truncation mutation, *ddc2-ΔC*, abolishes Mec1-Ddc2 interaction, thereby disrupting Ddc2 function [7]. The modified two-hybrid assay depicted the *ddc2-ΔC* mutation accurately (Fig. 2). We further explored whether the N-terminus of Ddc2 is required for Mec1-Ddc2 interaction or DNA damage tolerance. We found that the N-terminus of Ddc2 is dispensable for Ddc2 functions; both the *ddc2-ΔN1* and *ddc2-ΔN2* constructs supported Mec1-Ddc2 interaction and DNA damage tolerance in the modified two-hybrid system (Fig. 2). However, the *ddc2-ΔN3* mutation disrupted Mec1-Ddc2 interaction and thereby impaired proper DNA damage responses (Fig. 2). Thus, the middle and C-terminal regions of Ddc2 (amino acid 141–747) are essential for Mec1-Ddc2 complex formation and proper DNA damage responses.

### Isolation of the separation-of-function *ddc2-S4* mutation by using two-hybrid systems

**Figure 3:**
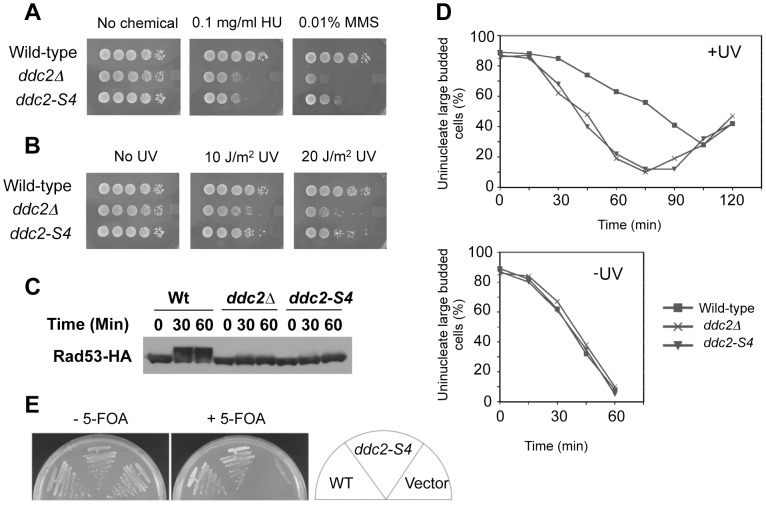
DNA damage response and proliferation of *ddc2-S4* mutants. (A, B) DNA damage sensitivity of *ddc2-S4* mutants. Serial dilutions of cultures were spotted on YEPD medium without or with MMS or HU (A). Cultures spotted on YEPD medium were irradiated with UV light (B). Plates were incubated at 30°C for two-three days. Strains used were wild-type (KSC1178), *ddc2Δ* (KSC1234) and *ddc2-S4* (KSC3153). (C) Effect of *ddc2-S4* on DNA damage checkpoint signaling. Wild-type (KSC1178), *ddc2Δ* (KSC1234), and *ddc2-S4* (KSC3153) cells expressing Rad53-HA were grown to log-phase and exposed to MMS (0.05%) at 30°C for the indicated length of time. Cells were harvested and subjected to immunoblotting analysis with anti-HA antibodies. (D) G2/M-phase DNA damage checkpoint in *ddc2-S4* mutants. Wild-type (KSC1178), *ddc2Δ* (KSC1234) or *ddc2-S4* (KSC3153) cells were arrested with nocodazole and irradiated or not irradiated with UV (50 J/m^2^). At the indicated times after release of UV-irradiated (+UV) and unirradiated (−UV) cultures from nocodazole, the percentage of uninucleate large budded cells was scored by DAPI staining. (E) Effect of *ddc2-S4* mutation on cell proliferation in the presence of *SML1*. *ddc2Δ* mutants carrying the *URA3*-marked YCp-DDC2 plasmid (KSC3308) were transformed with YCpT-DDC2, YCpT-DDC2-S4 or the control vector. Transformants were streaked and grown on plates containing medium with or without 5-fluoroorotic acid (5-FOA) at 30°C. Only cells that have lost the *URA3*-marker plasmid can proliferate in the presence of 5-FOA [62].

The above information prompted us to screen separation-of-function mutations within the amino residues (141–747) in the middle and C-terminal regions of Ddc2. We combined two different two-hybrid systems to isolate *ddc2* separation-of-function mutations that impair DNA damage responses but do not affect Mec1 localization to DNA damage sites. Proper Mec1 localization depends on Mec1-Ddc2 complex formation [11]. In the first screening, we adopted the functional two-hybrid assay as described above (see Fig. 2). After *in vitro* mutagenesis in *DDC2* of the AD-DDC2 construct, we searched for clones that maintain Mec1-Ddc2 interaction but fail to rescue the *ddc2Δ* mutation. The Mec1-Ddc2 complex accumulates at sites of DNA damage by interacting with RPA [9]–[11]. RPA consists of three subunits: Rfa1, Rfa2 and Rfa3 in budding yeast [44]. We have shown that the Mec1-Ddc2 complex interacts with Rfa1 or Rfa2 in a two-hybrid system [11]. In the second screening, we further selected clones that retain the interaction with Rfa1 in a two-hybrid system. After the second screening each mutant allele was fused to its own promoter and introduced into its own chromosome locus. One mutation, named *ddc2-S4*, conferred noteworthy sensitivity to DNA damaging agents (Fig. 3A and 3B). We therefore characterized the *ddc2-S4* mutation further. The *ddc2-S4* allele contains two substitution mutations; K263E and H382Y, both of which are required to exhibit full DNA damage sensitivity (data not shown).

We examined the effect of *ddc2-S4* mutation on checkpoint signaling in response to DNA damage (Fig. 3C). Checkpoint activation is correlated to the phosphorylation status of Rad53, a kinase that acts downstream of Mec1 [4]. Wild-type, *ddc2Δ* or *ddc2-S4* cells expressing HA-tagged Rad53 protein (Rad53-HA) were incubated with MMS, and subjected to immunoblotting analysis with anti-HA antibody. Rad53 was phosphorylated in wild-type cells after MMS treatment while no apparent phosphorylation was detected in *ddc2Δ* mutants. As found in *ddc2Δ* mutants, Rad53 phosphorylation was markedly decreased in *ddc2-S4* mutants. We examined whether *ddc2-S4* mutants are defective in DNA damage checkpoints. We first examined the G2/M-phase DNA damage checkpoint by monitoring mitotic division following UV irradiation (Fig. 3D). When cell cultures were released from nocodazole arrest following UV irradiation, wild-type cells exhibited delayed nuclear division while *ddc2-S4* cells underwent mitosis similar to *ddc2Δ* cells. Similarly, *ddc2-S4* mutant cells progressed faster than wild-type cells through the G1/S transition and S phase following DNA damage (Fig. S2 and Fig. S3). *DDC2* is indispensable for cell proliferation [5]–[7] but the *ddc2-S4* mutation does not perturb the essential function of Ddc2. Cells carrying the *ddc2-S4* mutation proliferated as well as those carrying the wild-type *DDC2* gene (Fig. 3E).

### Effect of *ddc2-S4* mutation on Mec1-Ddc2 complex formation and Mec1 localization to sites of DNA damage

**Figure 4:**
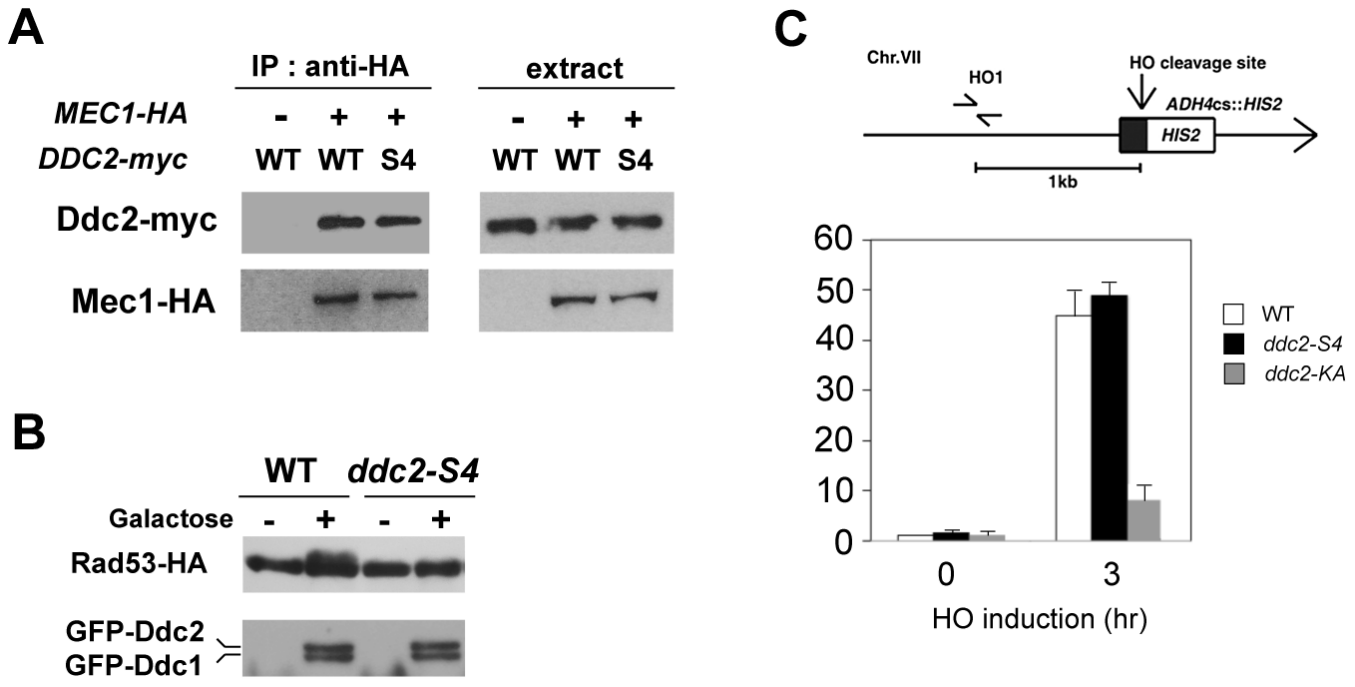
Effect of *ddc2-S4* on Mec1-Ddc2 interaction and Mec1 localization. (A) Effect of *ddc2-S4* mutation on Mec1-Ddc2 interaction. *MEC1-HA ddc2Δ* (KSC1340) or *ddc2Δ* (KSC1234) cells were transformed with YCp-DDC2-myc or YCp-DDC2-S4-myc. Extracts prepared from cells were subjected to immunoprecipitation with anti-HA antibodies. Immunoprecipitates and whole extracts were analyzed by immunoblotting with anti-HA or anti-myc antibodies. (B) Effect of *ddc2-S4* or *ddc2-KA* mutation on Mec1 localization to an HO-induced DSB. Wild-type (KSC1635), *ddc2-S4* (KSC2158) or *ddc2-KA* (KSC2159) cells expressing Mec1-HA were transformed with the YCpA-GAL-HO plasmid. Transformed cells were grown in sucrose and treated with nocodazole. After arrest at G2/M, the culture was incubated with galactose for 3 hr to induce HO expression, while half of the culture was maintained in sucrose to repress HO expression. (Top) The strains contain an HO cleavage site, marked with *HIS2*, at the *ADH4* locus on chromosome (Chr.) VII. The HO1 primer pair amplifies a region 1 kb away from the HO cleavage site. An arrow represents the telomere. (Bottom) Cells were subjected to chromatin immunoprecipitation with anti-HA antibodies. Association of Mec1 with an HO-induced DSB was analyzed by real-time PCR. Relative enrichment was determined from three independent experiments. (C) Effect of *ddc2-S4* on Rad53 phosphorylation after co-localization of Ddc1-LacI and Ddc2-LacI to a LacO array. Cells containing the LacO_256_ array and *RAD53-HA* with the combination of DDC1-LacI-GFP and DDC2-LacI-GFP (CBY88) or DDC2-S4-LacI-GFP (KSC2419) under the control of GAL promoter were grown in sucrose and arrested in nocodazole for 2 hr. Galactose was pulsed for 2 hr, and then cells were further incubated with glucose. Samples were collected at 2 hr after addition of glucose and subjected to immunoblotting analysis with anti-HA or anti-GFP antibodies.

We investigated the effect of *ddc2-S4* mutation on Mec1 localization to DNA damage sites. As discussed above, Mec1 localization depends on Mec1-Ddc2 complex formation [11]. We first examined the effect of *ddc2-S4* mutation on Mec1-Ddc2 interaction (Fig. 4A). Extracts were prepared from cells expressing Mec1-HA and Ddc2-myc or Ddc2-S4-myc and analyzed by immunoprecipitation with anti-HA antibodies. The immunoprecipitates were then probed with antibodies against the HA and myc epitopes. Co-precipitation of Ddc2 with Mec1 was detected only in extracts from cells expressing both Mec1-HA and Ddc2-myc. Ddc2 and Ddc2-S4 were similarly precipitated with Mec1, indicating that the *ddc2-S4* mutation does not impair Mec1-Ddc2 complex formation.

We next compared Mec1 association with HO-induced DSBs in wild-type and *ddc2-S4* mutants by chromatin immunoprecipitation (ChIP) assay (Fig. 4B). In budding yeast, HO endonuclease introduces a sequence-specific DSB. We used an experimental system in which cells contain a single HO cleavage site at the *ADH4* locus, and HO is expressed from the GAL-HO plasmid after incubation with galactose [11]. Cells expressing Mec1-HA were transformed with the GAL-HO plasmid. Transformed cells were grown initially in sucrose to repress *HO* expression, and then transferred to medium containing nocodazole to arrest at G2/M. After arrest, galactose was added to induce *HO* expression. Cells were then subjected to the ChIP assay. Mec1 associated with HO-induced DSBs in *ddc2-S4* mutants as efficiently as in wild-type cells. These results show that the *ddc2-S4* mutation does not impair Mec1 localization to sites of DNA damage. We previously showed that the substitution mutation (K177A, K178A) within the KKRK sequence (amino acids 177–180), *ddc2-KA*, confers DNA damage sensitivity similar to the *ddc2Δ* mutation but does not affect Mec1-Ddc2 complex formation [7]. We wondered whether the *ddc2-KA* mutation behaves like the *ddc2-S4* mutation. However, association of Mec1 with DSBs was significantly decreased in *ddc2-KA* cells compared with that in wild-type cells (Fig. 4B). Thus, the basic amino acid residues at the middle region of Ddc2 are required for efficient Mec1 localization to sites of DNA damage.

Tethering of multiple Ddc1 and Ddc2 proteins on chromatin stimulates Mec1-mediated checkpoint activation in the absence of DNA damage [31]. In this system an array of *lac* operator repeats (LacO_256_) was integrated into the genome and Ddc1 and Ddc2 were fused to *lac* repressor (LacI). Therefore, Ddc1- or Ddc2-LacI can be co-localized on chromatin through DNA binding activity of LacI. In turn, Ddc2-LacI can recruit Mec1 to the LacO repeat. We used this artificial tethering system to determine whether the *ddc2-S4* mutation is defective in activation of the Mec1-dependent signaling. Co-expression of Ddc1-LacI and Ddc2-LacI promoted Rad53 phosphorylation as found previously (Fig. 4C). By contrast, Rad53 phosphorylation was significantly decreased in cells expressing Ddc1-LacI and Ddc2-S4-LacI (Fig. 4C). These results are consistent with the idea that the *ddc2-S4* mutation causes defects in checkpoint activation but not in Mec1 localization.

### Effect of *ddc2-S4* mutation on Mec1 activation after DNA damage

**Figure 5:**
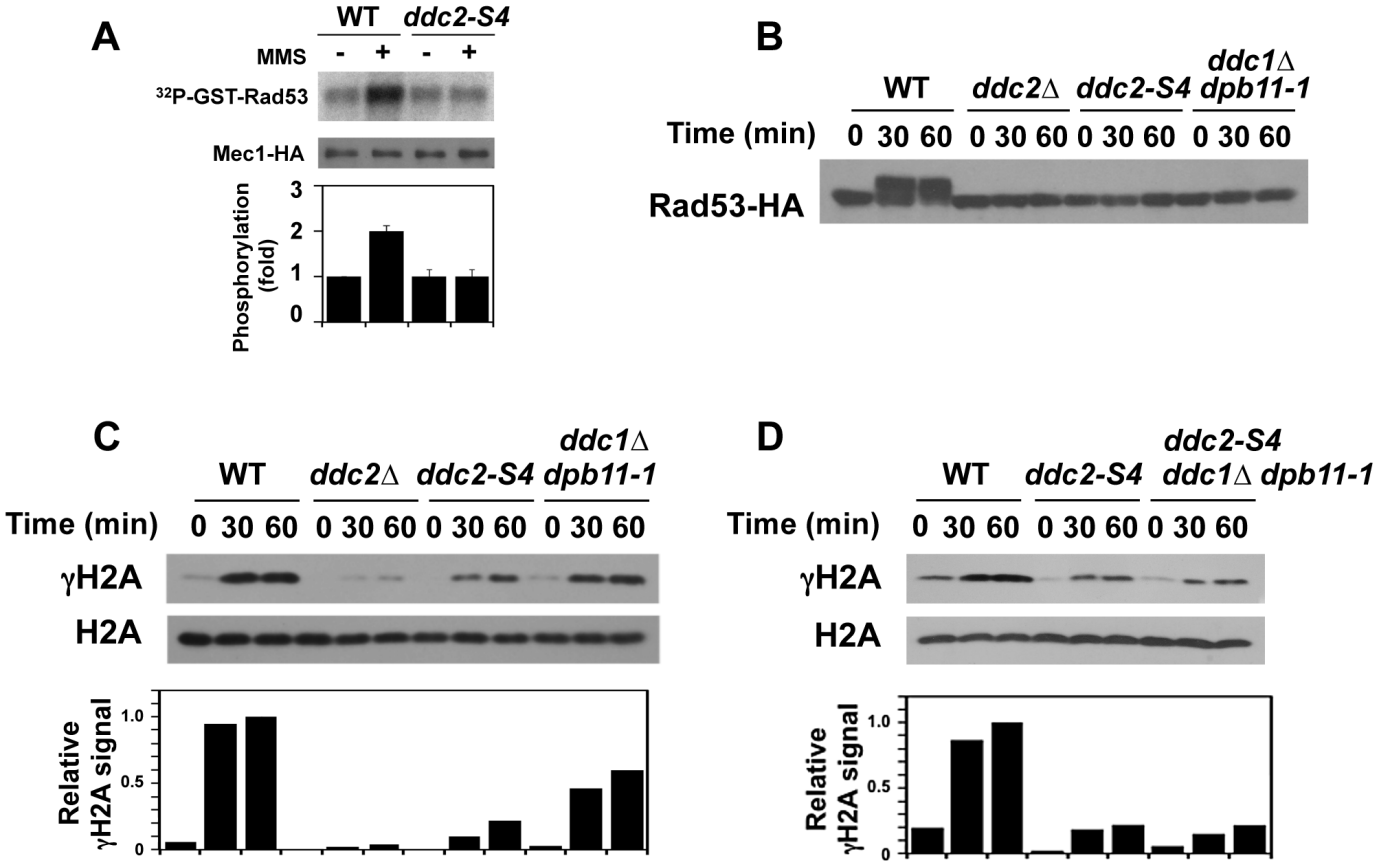
Effect of *ddc2-S4* on Mec1 activation after DNA damage. (A) Effect of *ddc2-S4* mutation on Mec1 activation. Wild-type (KSC1635) and *ddc2-S4* (KSC2158) cells expressing Mec1-HA protein were subjected to the *in vitro* kinase assay as in Fig. 1. (B) Effect of *ddc2-S4* or *ddc1Δ dpb11-1* mutation on Rad53 phosphorylation at G2/M. Wild-type (KSC1178), *ddc2Δ* (KSC1234), *ddc2-S4* (KSC3153) or *ddc1Δ dpb11-1* (KSC3190) cells expressing Rad53-HA were grown at 25°C and arrested with nocodazole at G2/M. Cells were then exposed to 0.05% MMS. Cells were collected at the indicated time point, and extracts were subjected to immunoblotting analysis with anti-HA antibodies. (C) Effect of *ddc2-S4* or *ddc1Δ dpb11-1* mutation on histone H2A phosphorylation at G2/M. The same extracts used in B were analyzed by immunoblotting with anti-phospho S129 (γH2A) or anti-H2A antibodies. (D) Histone H2A phosphorylation in *ddc2-S4* single and *ddc2-S4 ddc1Δ dpb11-1* triple mutants at G2/M. Wild-type (KSC1178), *ddc2-S4* (KSC3153) or *ddc2-S4 ddc1Δ dpb11-1* (KSC3244) cells were grown at 25°C and arrested with nocodazole at G2/M. Cells were then exposed to 0.05% MMS. Cells were collected at the indicated time point, and extracts were analyzed as in C.

We examined the effect of *ddc2-S4* mutation on Mec1 kinase activity (Fig. 5A). Wild-type and *ddc2-S4* cells expressing Mec1-HA protein were arrested with nocodazole at G2/M and then treated with MMS. The *ddc2-S4* mutation did not affect basal Mec1 kinase activity but ablated the increase in kinase activity after MMS treatment.

As shown above, Mec1 activation occurs even in *ddc1Δ dpb11-1* mutants at G2/M (see Fig. 1B). To further dissect Mec1 activation mechanisms, we monitored damage-induced phosphorylation of H2A and Rad53 in wild-type, *ddc2Δ*, *ddc2-S4* and *ddc1Δ dpb11-1* mutant cells at G2/M (Fig. 5B and 5C). We note that the *sml1Δ* mutation does not affect the cell proliferation of *ddc1Δ dpb11-1* mutant cells (Fig. S4). Cells expressing Rad53-HA were arrested at G2/M and then treated with MMS. Extracts from cells were subjected to immunoblotting analysis with anti-HA, anti-phospho H2A or anti-H2A antibodies. Rad53 was phosphorylated in wild-type cells after MMS treatment while phosphorylation was markedly decreased in *ddc2-S4* mutants as found in *ddc2Δ* or *ddc1Δ dpb11-1* mutants. Thus, *ddc2-S4* mutants are as defective in Rad53 phosphorylation as *ddc2Δ* or *ddc1Δ dpb11-1* mutants at G2/M. Histone H2A is phosphorylated after MMS treatment and its phosphorylation largely depends on Mec1 function [12]. Consistently, histone H2A was robustly phosphorylated in wild-type cells after MMS treatment but only faint phosphorylation was observed in *ddc2Δ* mutants. Phosphorylation was reduced in *ddc1Δ dpb11-1* mutants compared with that in wild-type cells [36], supporting the current view that Ddc1 and Dpb11 activate Mec1 [34], [35]. As discussed above, Mec1 localizes to sites of DNA damage in *ddc2-S4* mutants (see Fig. 4B) but not in *ddc2Δ* mutants [11]. Accordingly, histone H2A was phosphorylated more extensively after MMS treatment in *ddc2-S4* mutants than in *ddc2Δ*mutants. However, the level of H2A phosphorylation in *ddc2-S4* mutants was much lower than that in *ddc1Δ dpb11-1* mutants. Ddc1 or Dpb11 could activate Mec1 at sites of DNA damage in *ddc2-S4* mutants. We therefore examined the effect of *ddc1Δ dpb11-1* mutation on H2A phosphorylation in *ddc2-S4* mutants after MMS treatment (Fig. 5D). The *ddc2-S4 ddc1Δ dpb11-1* triple mutation did not further decrease H2A phosphorylation compared to the *ddc2-S4* single mutation. Thus, the *ddc2-S4* mutation causes more significant defects in Mec1 activation than the *ddc1Δ dpb11-1* mutation. These results support the idea that Ddc2 mediates Mec1 activation by a different mechanism than Ddc1 or Dpb11.

## Discussion

Previous studies have established that Ddc1 and Dpb11 directly activate Mec1 and play a key role in checkpoint activation at G1 and G2/M [34]–[36], [38]–[40]. In this study, we provided evidence suggesting that Ddc2 promotes Mec1 activation after DNA damage through a Ddc1- or Dpb11-independent mechanism at G2/M. Damage-induced Mec1 activation occurs even in *ddc1Δ dpb11-1* double mutants, but the *ddc2Δ* mutation abolishes Mec1 activation. Ddc2 recruits Mec1 to sites of DNA damage [8]–[11]. To gain more insight into the mechanism, we isolated and characterized the *ddc2-S4* mutation that confers defects in Mec1 activation but does not affect Mec1 recruitment. While Rad53 phosphorylation is similarly decreased in *ddc2-S4* and *ddc1Δ dpb11-1* mutants at G2/M, *ddc2-S4* mutants are more defective in phosphorylation of histone H2A than *ddc1Δ dpb11-1* mutants. These findings are consistent with the view that Ddc2 plays a pivotal role in not only Mec1 recruitment but also Mec1 activation after DNA damage.

We have shown that the middle and C-terminal regions of Ddc2 is critical for DNA damage recognition, Mec1-Ddc1 interaction and Mec1 activation. The results from the modified two-hybrid assay indicated that the middle and C-terminal regions are required for Mec1-Ddc2 interaction and proper DNA damage responses. Characterization of the *ddc2-S4* mutation, consisting of two mutations (K263E and H382Y), revealed that these regions are involved in Mec1 activation as well. Similarly, the middle and C-terminal regions of human and *Xenopus* ATRIP are involved in DNA damage recognition, ATR-ATRIP complex formation and checkpoint activation [45]–[49]. Thus, although not structurally conserved, the middle and C-terminal regions of ATRIP family proteins share crucial functions. The middle region of ATRIP or Ddc2 also mediates TopBP1 or Dpb11 interaction [38], [49]. It is not determined, however, whether Ddc1 or Dna2 activates Mec1 by interacting with the middle region of Ddc2. The N-terminal region consists of the checkpoint protein recruitment domain (CRD) and the coiled-coil (CC) domain [46], [50]–[52]. Previous studies have shown that the ATRIP/Ddc2 CRD mediates efficient interaction of ATR/Mec1 with RPA [52], while other studies have found that the N-terminus of ATRIP is important but not essential for the interaction with RPA-coated ssDNA [46], [48]. The CRD deletion mutation in *DDC2* was found to impede Mec1 localization and sensitize cells to DNA damaging agents [52]. We found that the N-terminal region of Ddc2 is dispensable for proper DNA damage responses; N-terminal truncated Ddc2-ΔN2 (141–747) protein, expressed from its own promoter, also restored DNA damage resistance to *ddc2Δ* mutants (data not shown). Our results, however, do not exclude the possibility that the N-terminus of Ddc2 contributes to Mec1 localization to sites of DNA damage. Alternatively, we found that the *ddc2-KA* mutation at the middle region confers defects in Mec1 localization to DNA lesions although the *ddc2-KA* mutation does not disrupt Mec1-Ddc2 interaction [7]. Thus, multiple regions in ATRIP/Ddc2 appear to control ATR/Mec1 localization to sites of DNA damage. Studies using human cells identified that the CC domain is important for the cellular response to DNA damage or replication stress [50], [51]. We showed that the CC domain of Ddc2 is dispensable for DNA damage responses. In *Xenopus*, the CC domain of ATRIP is dispensable for Chk1 activation as well [46]. Thus, the CC domain of ATRIP family proteins is structurally similar but not functionally conserved.

We have provided evidence indicating that Ddc2 promotes Mec1 activation through a Ddc1- or Dpb11-independent mechanism. Previous studies have established that Ddc1 and Dpb11 have crucial functions in G1- and G2/M-DNA damage checkpoints by increasing the catalytic activity of Mec1 [34]–[36], [38], [39]. Thus, several different mechanisms increase the catalytic activity of Mec1 in response to DNA damage. Our *in vitro* kinase assay using immunoprecipitated Mec1 protein was not able to detect Ddc1- or Dpb11-dependent Mec1 activation. One explanation is that Mec1 does not interact stably with Ddc1 or Dpb11 after immunoprecipitation. Previous studies show that the addition of purified Dpb11 protein increases the catalytic activity of immunoprecipitated Mec1 [38], [40]. Co-immunoprecipitation of Ddc1 with Mec1 has not been reported so far.

Activation of the downstream kinase Rad53 requires its interaction with Rad9 at sites of DNA damage [20], [21]. Mec1 phosphorylates histone H2A to promote Rad9 localization to sites of DNA damage [12], [14], [15]. Histone H2A is phosphorylated more strongly in *ddc1Δ dpb11-1* mutants than in *ddc2-S4* mutants whereas Rad53 phosphorylation is similarly defective in *ddc1Δ dpb11-1* and *ddc2-S4* mutants. These observations are consistent with the current model in which Ddc1 and Dpb11 are also involved in checkpoint activation events that operate downstream of Mec1 activation. Previous studies have shown that the Ddc1-Mec3-Rad17 complex contributes to Rad9 recruitment [53] and Dpb11 binds Rad9 to positively regulate Rad53 activation [40], [54]. Introduction of the *ddc1Δ dpb11-1* double mutation does not further decrease histone H2A phosphorylation in *ddc2-S4* mutants. One likely explanation is that Ddc2 and Ddc1/Dpb11 stimulate Mec1 sequentially; that is, Ddc2-dependent activation precedes Ddc1- and Dpb11-dependent activation although we cannot exclude the possibility that Ddc2-S4 protein is defective for the interaction with Ddc1 and Dpb11. Recent studies show that human ATR undergoes auto-phosphorylation at damage sites and thereby promotes ATR-TopBP1 interaction [55]. Notably, ATR autophosphorylation depends on ATRIP but not on TopBP1 function [55]. As Ddc2 mediates Mec1 activation in a Ddc1- or Dpb11-independent manner, ATRIP might stimulate ATR independently of TopBP1 at sites of DNA damage in human cells.

Tethering of Ddc2 and Ddc1 protein on chromosomal DNA leads to Rad53 phosphorylation in the absence of DNA damage [31], but the *ddc2-S4* mutation was found to abolish Rad53 phosphorylation. Co-localization of Mec1 and Mrc1 can trigger checkpoint activation in the absence of DNA damage as well, but this Mec1- and Mrc1-mediated checkpoint activation is independent of both Ddc1 and Dpb11 function [56]. Given that the *ddc2-S4* mutation is defective in Mec1 activation, these results are consistent with the view that the congregation of Mec1-Ddc2 complexes turns on the Ddc2 function that increases the catalytic activity of Mec1. Since the tethering system triggers checkpoint responses in the absence of DNA damage [31] [56], the interaction of the Mec1-Ddc2 complex with RPA might be dispensable for Mec1 activation. However, tethered Mec1-Ddc2 protein could contact with chromosomal DNA. Studies using *Xenopus* extracts have suggested that DNA substrates with specific structures can activate ATR-ATRIP in the absence of RPA [46]. Further experiments will be aimed at elucidating the more precise biochemical properties of the Mec1-Ddc2 complex in the presence or absence of RPA and DNA.

## Materials and Methods

### Plasmids and strains

DNA fragments containing the *DDC2* gene were amplified by the primer pair (KS437 and KS1603, KS437 and KS1604, or KS437 and KS1605), digested with BamHI and SalI, and cloned into pGAD-C1 [57], generating pGAD-DDC2-ΔN1, pGAD-DDC2-ΔN2 or pGAD-DDC2-ΔN3, respectively. The pGAD-DDC2-ΔC plasmid was constructed as follows. The *ddc2-ΔC* (*pie1-ΔC*) mutation [7] was amplified by PCR, digested with BamHI and SalI and cloned into pGAD-C1, generating pGAD-DDC2-ΔC. *PIE1* is an alias of the *DDC2* gene [7]. The *RFA1* gene was amplified by PCR using the primer pair (KS882 and KS883), digested with BamHI and SalI and cloned into pGBD-C1 [57], creating pGBD-RFA1. To generate YCp-DDC2-myc, an EcoRI-SalI fragment of YCpT-DDC2-myc [7] was cloned in YCplac33 [58]. The pGBD-MEC1(2-2368), pGAD-DDC2, and YCpT-RAD53-HA plasmids were described before [7], [11]. The isolated *ddc2* mutations were transferred from pGAD-DDC2 to YCp-DDC2-myc as follows. Each mutation was amplified by PCR using the oligonucleotide pair KS1603 and KSX001. The resulting PCR fragments were treated with XbaI and SalI, cloned into XbaI-SalI-digested YCp-DDC2-myc. YCp-DDC2-S4-myc is a version containing the *ddc2-S4* mutation. The SphI-SpeI fragment from the *ddc2-S4* allele was introduced into pJAM150 [31], YCp-DDC2 [7] or YCpT-DDC2 [7], resulting in pGFP-DDC2-S4-LacI, YCp-DDC2-S4 or YCpT-DDC2-S4, respectively. The GFP-DDC2-S4-LacI cassette was introduced into the *DDC2* locus after digestion with NheI. The *ddc2-S4* or *ddc2-KA* (*pie1-KA*) mutation [7] was fused to the *URA3* marker by PCR using the KS2047 and KS2048 oligonucleotides [59] and integrated into the *DDC2* locus. The *ddc1Δ* mutation has been described [24]. Deletion of *DDC2* or *SML1* was generated by a PCR-based method [60], [61]. The *dpb11-1* mutation [43] was obtained from Dr. H. Araki (National Institute of Genetics, Mishima, Japan). All of the strains and oligonucleotides used in this study are listed in Tables 1 and 2, respectively.

**Table 1:** Strains used in this study.

Strain	Genotype	
KSC1635	*MAT*a-*inc*	*MEC1-2HA::TRP1 sml1Δ::LEU2 ADH4cs::HIS2*
KSC1645	*MAT*a-*inc*	*mec1-KN-2HA::TRP1::URA3 sml1Δ::LEU2 ADH4cs::HIS2*
KSC1333	*MAT*a	*MEC1-2HA::TRP1*
KSC3130	*MAT*a	*MEC1-2HA::TRP1 ddc1Δ::LEU2 dpb11-1*
KSC1636	*MAT*a-*inc*	*MEC1-2HA::TRP1 ddc2Δ::LEU2 sml1Δ::LEU2 ADH4cs::HIS2*
KSC2158	*MAT*a-*inc*	*MEC1-2HA::TRP1 ddc2-S4::URA3 sml1Δ::LEU2 ADH4cs::HIS2*
KSC2159	*MAT*a-*inc*	*MEC1-2HA::TRP1 ddc2-KA::URA3 sml1Δ::LEU2 ADH4cs::HIS2*
KSC1178	*MAT*a	*sml1Δ::LEU2*
KSC1234	*MAT*a	*ddc2Δ::LEU2 sml1Δ::LEU2*
KSC1340	*MAT*a	*MEC1-2HA::TRP1 ddc2Δ::LEU2 sml1Δ::LEU2*
KSC3153	*MAT*a	*ddc2-S4::URA3 sml1Δ::LEU2*
KSC3190	*MAT*a	*ddc1Δ::LEU2 dpb11-1 sml1Δ::LEU2*
KSC3244	*MAT*a	*ddc2-S4::URA3 ddc1Δ::TRP1 dpb11-1 sml1Δ::LEU2*
*KSC3131*	*MAT*a	*ddc1Δ::LEU2 dpb11-1*
*KSC3308*	*MAT*a	*ddc2Δ::LEU2* [YCp-DDC2]
PJ69-4A	*MAT*a	*GAL2-ADE2 GAL1-HIS3*
KSC2077	*MAT*a	*GAL2-ADE2 GAL1-HIS3 ddc2Δ::TRP1 sml1Δ::KanMX*
CBY51	*MAT*a	*GAL-DDC1-GFP-LacI::URA3 RAD53-HA::LEU2 LacO_256_::TRP1*
CBY88	*MAT*a	*GAL-DDC1-GFP-LacI::URA3 GAL-DDC2-GFP-LacI::HIS3 RAD53-HA::LEU2 LacO_256_::TRP1*
KSC2419	*MAT*a	*GAL-DDC1-GFP-LacI::URA3 GAL-DDC2-S4-GFP-LacI::HIS3 RAD53-HA::LEU2 LacO_256_::TRP1*

All the KSC strains, except for KSC2077 or KSC2419, are isogenic and derived from KSC006 (*MAT*a *ade1 his2 leu2 trp1 ura3*) [7]. KSC2419 or KSC2077is derived from CBY51 or PJ69-4A, respectively. PJ69-4A has been described [7]. CBY51 and CBY88 have been obtained from David Toczyski [31]. *MAT*a-*inc* is a mutation of the HO cleavage site [10].

**Table 2:** List of oligonucleotides used in this study.

Name	Sequence (5′ – 3′)
KS437	CTCGTCGACCTTACAGTCCCATTGAGAT
KS882	CTAGGATCCATGAGCAGTGTTCAACTTTCGAGGGGC
KS883	CTAGTCGACTTAAGCTAACAAAGCCTTGGATAACTC
KS1603	ATTGGATCCACTTTGGAGGTTACAACGACCAC
KS1604	GAAGGATCCACGAATGTAAAACCACCGTCAAC
KS1605	CATGGATCCATAGGCGCTGACCTGAGCAC
KS2047	CGAGCTCGAATTCATCGATTACAGTCCCATTGAGATATATA
KS2048	ATTACAAGGTTTCTATAAATCGTTGACATTTTCCCCTTTTGATTGTTGCCG TGTCACCATGAACGACAATTC
KSX001	AGCGGATAACAATTTCACACAGGA

### Two-hybrid screening

DNA fragments encompassing the middle and C-terminal regions of Ddc2 (amino acid141 to 747) were amplified by error prone PCR in the presence of 0.2 mM MnCl_2_. The resulting PCR products were introduced with XbaI-SalI digested pGAD-DDC2 into the two-hybrid tester *ddc2Δ sml1Δ* strain (KSC2077) transformed with pBD-MEC1(2-2368). Transformants were plated on selection medium containing 1 mM 3-aminotriazole (AT) and allowed to grow for 3 days. Colonies on AT were then replica-plated on medium containing 1 mg/ml of HU. Plasmids were recovered from the colonies that did not grow on medium containing HU and retested. Out of ∼10,000 transformants, ten plasmids were found to support proliferation on medium containing AT but not in the presence of HU. Those plasmids were further introduced into the PJ69-4A strain [57] carrying pBD-RFA1. Eight plasmids were found to support Ddc2-Rfa1 interaction. The *ddc2-S4* mutation expressed from the own promoter still conferred notable sensitivity to HU and MMS.

### Mec1 kinase assay

Cells were grown at 25°C and arrested with nocodazole for 3 hr. Cells were then treated with 0.05% MMS for different length of times. One hundred OD_600_ cells expressing HA-tagged Mec1 protein were harvested and disrupted in the lysis buffer [20 mM HEPES-KOH pH 7.5, 100 mM NaCl, 0.1% TritonX-100, 1 mM EDTA pH 8.0] containing 15 mM PNPP, 1 µg/ml leupeptin, 1 µg/ml pepstatin, 1 mM sodium orthovanadate, 1 mM PMSF by bead beating with Multi-beads shocker (Yasui Kikai, Osaka, Japan) at 4°C. HA-tagged Mec1 was immunoprecipitated as described previously [61]. Kinase reactions were initiated by addition of 1 µg of gluthatione S-transferase (GST)-Rad53 to immunoprecipitates in 40 µl of the reaction buffer (20 mM Hepes-KOH [pH 7.5], 10 mM MgCl_2_, 4 mM MnCl_2_, 50 µM ATP) containing 5 µCi [γ-^32^P] ATP (3,000 Ci/mmol). After 10 min of incubation at 30°C, the reaction was terminated by the addition of 10 µl of 5× loading dye [61]. The reaction mixtures were separated on SDS-polyacrylamide gels, and phosphorylation was quantified with a phosphorimager system (Typhoon 8600, GE Healthcare).

### Checkpoint activation by tethering of Ddc1 and Ddc2 proteins

Cells were treated with some modifications according to the method described previously [31]. LacO_256_ cells carrying Rad53-HA, GFP-Ddc1-LacI and GFP-Ddc2-LacI or GFP-Ddc2-S4-LacI were grown in 2% sucrose and 0.05% glucose. Cells were arrested by the incubation with nocodazole (final concentration 15 µg/ml) for 2 hr. Galactose (final concentration 2%) was added to one half culture to induce transcription of the LacI fusions for 2 hr. Cells were then incubated with glucose (final concentration 2%) to shut off transcription for 2 hr. After incubation with glucose, cells were harvested for immunoblotting analysis. All the cultures were incubated at 30°C.

### Other methods

Chromatin immunoprecipitation assay was carried out as described [11], [61]. The DNA damage sensitivity assay was determined as described [61]. Immunoblotting was described previously [61]. UV and MMS synchrony experiments were carried out as described previously [7], [27]. Plasmid shuffle with 5-fluoroorotic acid (5-FOA) was performed as described previously [62], [63]. Rabbit anti-GFP antibody (ab290) and anti-phospho histone H2A antibody (ab15083) were purchased from Abcam (Cambridge, MA). Rabbit anti-H2A antibody (39235) was obtained from Active Motif (Carlsbad, CA).

## Supporting Information

Figure S1 Flow cytometry analysis of nocodazole-arrested wild-type and *ddc1Δ dpb11-1* mutants cells. Wild-type (KSC1333) and *ddc1Δ dpb11-1* (KSC3130) cells expressing Mec1-HA protein were grown as in Figure 1A. Cells were collected before (− Noc) and after incubation with nocodazole (+ Noc). Cells were not exposed to MMS. Cells were examined for DNA content by flow cytometry. Dotted lines indicate the DNA content of 1C and 2C cells.
(TIFF)

Figure S2 G1-phase DNA damage checkpoint in *ddc2-S4* mutants. Wild-type (KSC1178), *ddc2Δ* (KSC1234) and *ddc2-S4* (KSC3153) cells were grown at 30°C, arrested with α-factor, and irradiated or not irradiated with UV light. The percentage of budded cells was scored at the indicated times after release of UV-irradiated (+UV) and unirradiated (−UV) cultures from α-factor.
(TIFF)

Figure S3 S-phase DNA damage checkpoint in *ddc2-S4* mutants. Wild-type (KSC1178), *ddc2Δ* (KSC1234) and *ddc2-S4* (KSC3153) cells were synchronized with α-factor in G_1_ and released in either the presence (+) or the absence (−) of 0.03% MMS at 30°C. Aliquots of cells were collected at the indicated times after release from α-factor treatment and examined for DNA content by flow cytometry. The top panels represent asynchronous (As) cells not treated with MMS at 30°C and are included as a reference. Dotted lines indicate the DNA content of 1C and 2C cells.
(TIFF)

Figure S4 Effect of *sml1Δ* mutation on the cell proliferation of *ddc1Δ dpb11-1* mutants. Wild-type (KSC006), *ddc1Δ dpb11-1* (KSC1234), *ddc1Δ dpb11-1 sml1Δ* (KSC1234) cells were serially diluted and spotted on plates containing rich medium. Plates were incubated at the indicated temperatures.
(TIFF)
